# Systemic inflammatory response syndrome and multiple organ dysfunction syndrome caused by acute mountain sickness: a case report and literature review

**DOI:** 10.3389/fphys.2025.1546307

**Published:** 2025-03-07

**Authors:** Bowen Wang, Mengjia Peng, Guoyong Kou, Fei Fang, Jinhang Gao

**Affiliations:** ^1^ Department of Gastroenterology, West China Hospital, Sichuan University, Chengdu, China; ^2^ Department of Emergency, General Hospital of Tibet Military Command, Lhasa, China; ^3^ Center of Endoscopy, General Hospital of Tibet Military Command, Lhasa, China

**Keywords:** acute mountain sickness, acute gastrointestinal bleeding, systemic inflammatory response syndrome, multiple organ dysfunction syndrome, oxygen therapy

## Abstract

Acute mountain sickness (AMS) is a common condition following rapid exposure to high altitude, though severe complications such as acute gastrointestinal bleeding, systemic inflammatory response syndrome (SIRS) and multiple organ dysfunction syndrome (MODS) are rare. Herein, we report a case of SIRS and MODS in a young traveler who visited Lhasa, Tibet (elevation 3,650 m). Three days after arrival, the patient developed headache, abdominal pain, significant hematemesis, and persistent hypotension. Gastroscopy revealed diffuse bleeding of the gastric mucosa. Laboratory tests indicated multi-organ dysfunction involving the lungs, liver, and kidneys. The patient responded well to conservative treatment of continuous oxygen supplementation. This case represents one of the first reported instances of acute gastric mucosal injury and MODS induced by AMS, underscoring the significant medical risks associated with high-altitude environments.

## Introduction

Acute Mountain Sickness (AMS) is a common condition affecting individuals ascending to high altitudes, typically manifesting as headache, nausea, and dizziness (Gatterer et al., 2024). While AMS is generally self-limiting, severe cases can lead to life-threatening complications such as High Altitude Pulmonary Edema (HAPE) and High Altitude Cerebral Edema (HACE) (Pena et al., 2022; Miglani et al., 2020). However, critical illnesses stemming from AMS, particularly those involving multiple organ dysfunction syndrome (MODS), are relatively rare and may often be misdiagnosed in clinical settings (Richalet et al., 2024; Meier et al., 2017). Here, we present a case of a young patient who developed SIRS and MODS following rapid ascent to Lhasa, Tibet, at an elevation of 3,650 m. Despite the rarity of such severe manifestations, our patient exhibited significant clinical symptoms including persistent hypotension, diffuse gastric mucosal hemorrhage. Meanwhile, we conducted a literature review about the MODS in the context of AMS.

## Case presentation

A 26-year-old male patient with normal body type presented to the emergency department of the General Hospital of Tibet Military Command (Lhasa, Tibet, China) on 5 August 2024, with a primary complaint of shortness of breath, headache and abdominal pain accompanied by hematemesis for 1 day. The patient arrived in Lhasa (average altitude 3,650 m) by airplane from Shanghai (average altitude 3 m) 3 days prior. One day before admission, he developed difficulty breathing, epigastric pain, and massive hematemesis. The abdominal pain was described as intermittent distension, and hematemesis occurred six times with large volumes each time. The patient denied any history of gastric disorders, acetaminophen and dexamethasone administration or alcohol consumption. Upon examination, vital signs revealed a body temperature of 36.8°C, pulse 141 beats per minute, respiratory rate 31 breaths per minute, and blood pressure 86/42 mmHg. Without supplemental oxygen, the patient’s blood oxygen saturation was 28% by blood gas analysis. Distinct cyanosis of the lips was observed. Auscultation of the lungs revealed coarse breath sounds with diffuse wet rales. Mild tenderness in the epigastric region was noted without rebound tenderness or muscle guarding.

**FIGURE 1:**
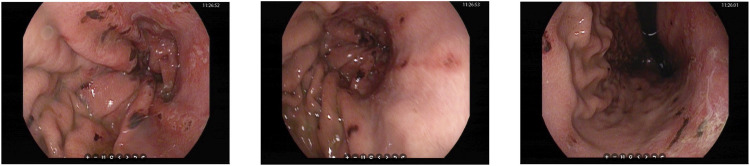
Gastroscopy images showing diffuse bleeding points on the gastric mucosa in the body of the stomach.

**FIGURE 2:**
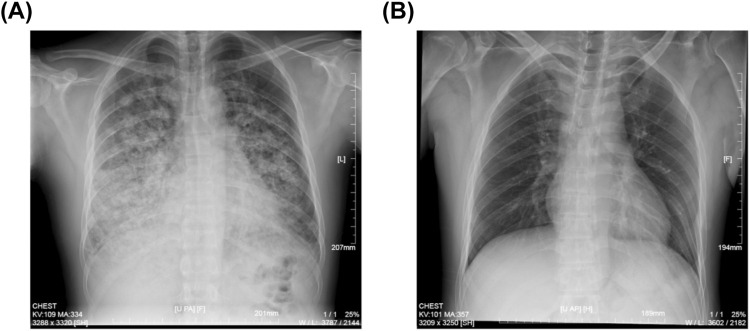
Chest X-ray images of the patient. **(A)** Spine position of chest X-ray image showing the HAPE at the patient’s admission to our hospital. **(B)** Upright position of chest X-ray image after continuous oxygen supplementation of 3 days.

An urgent gastroscopy was performed, revealing acute diffuse hemorrhagic gastritis (Figure 1). A chest X-ray suggested possible high-altitude pulmonary edema (Figure 2A). Laboratory tests indicated elevated white blood cell count (WBC) at 17.1 × 10^9^/L (normal range: 3.5–9.5 × 10^9^/L), decreased platelets (PLT) at 45 × 10^9^/L (normal range: 125–350 × 10^9^/L), increased C-reactive protein (CRP) at 38.1 mg/L (normal range: 0–10 mg/L), significantly elevated alanine aminotransferase (ALT) 3978 U/L (normal range: 9–50 U/L), aspartate aminotransferase (AST) 5687 U/L, normal range: 15–40 U/L), lactate dehydrogenase (LDH) 7825 U/L (normal range: 120–250 U/L), creatinine (Crea) at 348.7 μmol/L (normal range: 57–97 μmol/L), and Urea nitrogen (Urea) at 26.01 mmol/L (normal range: 3.1–8.0 mmol/L). Arterial blood gas analysis showed a partial pressure of oxygen (PaO_2_) of 55 mmHg (normal range: 83–108 mmHg). These laboratory findings suggested the presence of MODS, including impairment of pulmonary, hepatic, and renal functions.

**FIGURE 3:**
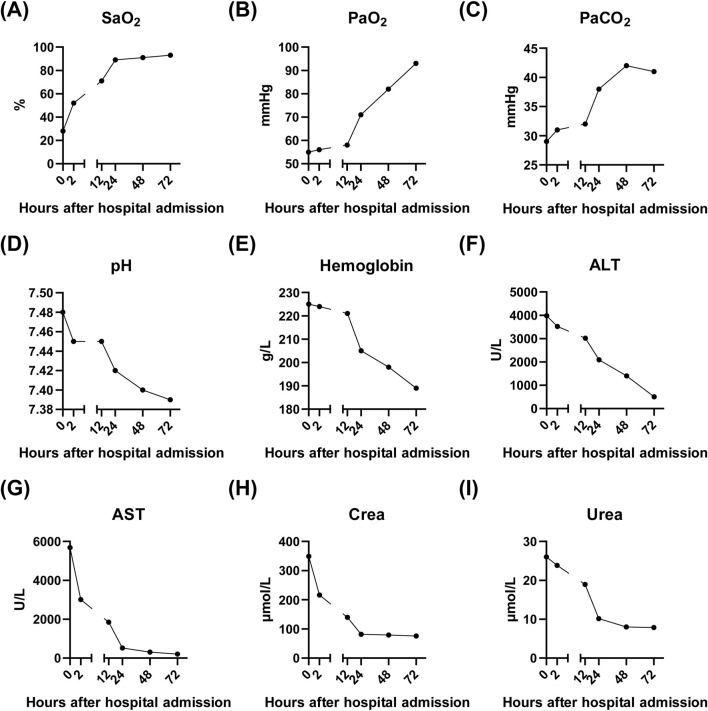
Laboratory results of the patient after admission to our hospital. **(A–E)** showing the recovery of lung function after continuous oxygen supplementation of 3 days. **(F–G)** showing the recovery of liver function after continuous oxygen supplementation of 3 days. **(H–I)** showing the recovery of kidney function after continuous oxygen supplementation of 3 days. SaO_2_, Arterial oxygen saturation; PaO_2_, Partial pressure of oxygen in arterial blood; PaCO_2_, Partial pressure of carbon dioxide in arterial blood; pH, Potential of hydrogen in arterial blood; ALT, Alanine aminotransferase; AST, Aspartate aminotransferase; Crea, Creatinine; Urea, Urea nitrogen.

The patient was immediately placed on continuous low-flow oxygen therapy (2–3 L/min). Following the initiation of oxygen therapy, the patient ceased hematemesis and the abdominal pain resolved. By the third day of hospitalization, fecal occult blood testing was negative. The patient’s respiratory distress improved significantly, and a follow-up chest X-ray on the third day demonstrated complete resolution of the pulmonary infiltrates (Figure 2B). Subsequent laboratory tests showed significant recovery in PaO_2_, PaCO_2_, pH, liver function tests, and renal function indicators (Figure 3).

## Literature review

AMS symptoms vary in severity and onset time, typically appearing within 6–24 h after reaching high altitudes above 2,500 m (Gatterer et al., 2024). Severe forms of AMS can progress to HACE, HAPE, and even MODS, all of which are medical emergencies requiring prompt descent and medical intervention (Simancas-Racines et al., 2018). Early recognition and appropriate management are crucial to prevent life-threatening complications (Luks et al., 2017).

Table 1 summarizes the key features of nine patients reported in the literature who developed MODS following AMS. Notably, these patients were predominantly young or even children. They presented with initial symptoms of AMS, which were often dismissed or underestimated. Over subsequent days, their condition deteriorated significantly, necessitating intensive care unit (ICU) admission. A striking observation was the frequent occurrence of pulmonary dysfunction among these patients, suggesting that hypoxia post-ascent plays a pivotal role in triggering MODS. Fortunately, these patients responded well to oxygen therapy, and their condition was rapidly brought under control.

**TABLE 1:** Clinical information of MODS caused by AMS (n = 9).

Patient no.	First author (publication year)	Country	Gender	Age (years)	Onset time after reaching high altitude areas (altitude)	Clinical manifestations	Affected organs	Treatment
1	Wei et al. (2009)	China	Female	20	2 days (>5,500 m)	Headache, dizziness, nausea, dyspnea and vomiting	Lung, brain, and kidney	Oxygen therapy and CRRT
2	Zhu et al. (2010)	China	Male	26	3 days (>3,000 m)	Headache, dyspnea and vomiting	Lung, liver, pancreas, and kidney	Oxygen therapy and proton pump inhibitor
3	Asseri et al. (2022)	Saudi Arabia	Female	9	12 h (2,200 m)	Fever, mild cough, and sore throat	Lung, heart, and liver	Oxygen therapy
4	Asseri et al. (2022)	Saudi Arabia	Male	11	1 days (3,015 m)	Dyspnea and cough	Lung and heart	Oxygen therapy and road-spectrum antibiotics
5	Asseri et al. (2022)	Saudi Arabia	Male	11	2 days (3,015 m)	Dyspnea and cough	Lung, heart, and liver	Oxygen therapy and road-spectrum antibiotics
6	Wang et al. (2024)	China	Male	21	3 days (3,845 m)	Fever, dyspnea, and cough	Lung, liver, and kidney	Oxygen therapy and angiotensin receptor blockers
7	Brar and Garg (2012)	India	Male	29	5 days (2,438 m)	Throbbing, headache with nausea and vomiting	Lung, brain, and kidney	Oxygen therapy and hydrocortisone
8	Gilbert-Kawai et al. (2016)	United Kingdom	Male	30	2 days (5,300 m)	Dizziness, dyspnea and vomiting	Lung, heart, and kidney	Oxygen therapy
9	Bhandari et al. (2017)	Nepal	Male	31	4 days (>3,000 m)	Dizziness, blurring of vision	Lung, brain, and eyes	Oxygen therapy

## Discussion

While AMS typically presents with headache, nausea, dizziness, fatigue, and sleep disturbances, severe cases can lead to complications such as gastrointestinal bleeding, SIRS and MODS. However, considering that AMS induced gastrointestinal ulcers and MODS are very rare, most clinics may focus more on the patient’s clinical symptoms (hematemesis, liver and kidney function damage, etc.) and overlook the patient’s medical history (Gatterer et al., 2024, Luks et al., 2017). Since oxygen therapy represents the cornerstone of AMS management, misdiagnosis and subsequent inappropriate treatment strategies may significantly compromise therapeutic efficacy.

The pathogenesis of AMS-induced gastrointestinal bleeding, SIRS and subsequent MODS involves a complex interplay of hypoxia, systemic inflammation, and oxidative stress (Pena et al., 2022, Wu et al., 2007a). Rapid ascent to high altitudes leads to reduced barometric pressure and oxygen availability, causing tissue hypoxia (Titz et al., 2024). This triggers compensatory mechanisms such as increased ventilation and heart rate, which, while attempting to enhance oxygen delivery, can exacerbate tissue hypoxia and contribute to organ dysfunction (Wilkins et al., 2015; Wu et al., 2007b; Wu and Liu, 2006). Concurrently, individuals ascending to elevated altitudes may encounter acid-base disturbances, notably respiratory alkalosis resulting in decreased PaCO_2_ levels. This physiological response could potentially serve as a pathogenic mechanism underlying acute gastric mucosal injury (Krishnan and Lotfollahzadeh, 2025). Furthermore, the possible influence of heightened adrenaline concentrations during stressful conditions must be considered, as it may also play a role in the development of gastric mucosal lesions (Guzman and Kruse, 1985).

Oxygen therapy is a cornerstone in the management of AMS and its complications, including gastrointestinal bleeding, SIRS and MODS (Schneider et al., 2024). By correcting hypoxia, oxygen therapy improves tissue oxygenation, reduces edema, and mitigates systemic inflammation (West, 2015). In patients with AMS-induced gastrointestinal bleeding, oxygen therapy can stabilize hemodynamic parameters, improve mucosal healing, and reduce the risk of further bleeding. For those progressing to MODS, oxygen therapy is still crucial in preventing organ failure and improving overall outcomes.

In conclusion, AMS-induced gastrointestinal bleeding and multiple organ dysfunction syndrome represent severe complications of high-altitude exposure that demand prompt recognition and aggressive management. This discussion underscores the importance of early diagnosis through meticulous clinical evaluation and emphasizes the lifesaving potential of timely oxygen therapy.

## Data Availability

The raw data supporting the conclusions of this article will be made available by the authors, without undue reservation.
